# Profiling the Mitochondrial Proteome of Leber’s Hereditary Optic Neuropathy (LHON) in Thailand: Down-Regulation of Bioenergetics and Mitochondrial Protein Quality Control Pathways in Fibroblasts with the 11778G>A Mutation

**DOI:** 10.1371/journal.pone.0106779

**Published:** 2014-09-12

**Authors:** Aung Win Tun, Sakdithep Chaiyarit, Supannee Kaewsutthi, Wanphen Katanyoo, Wanicha Chuenkongkaew, Masayoshi Kuwano, Takeshi Tomonaga, Chayanon Peerapittayamongkol, Visith Thongboonkerd, Patcharee Lertrit

**Affiliations:** 1 Department of Biochemistry, Faculty of Medicine Siriraj Hospital, Mahidol University, Bangkok, Thailand; 2 Medical Proteomics Unit, Office for Research and Development, Faculty of Medicine Siriraj Hospital, Mahidol University, Bangkok, Thailand; 3 Department of Ophthalmology, Faculty of Medicine Siriraj Hospital, Mahidol University, Bangkok, Thailand; 4 Laboratory of Proteome Research, National Institute of Biomedical Innovation, Osaka, Japan; 5 Center for Research in Complex Systems Science, Mahidol University, Bangkok, Thailand; Kunming Institute of Zoology, Chinese Academy of Sciences, China

## Abstract

Leber’s Hereditary Optic Neuropathy (LHON) is one of the commonest mitochondrial diseases. It causes total blindness, and predominantly affects young males. For the disease to develop, it is necessary for an individual to carry one of the primary mtDNA mutations 11778G>A, 14484T>C or 3460G>A. However these mutations are not sufficient to cause disease, and they do not explain the characteristic features of LHON such as the higher prevalence in males, incomplete penetrance, and relatively later age of onset. In order to explore the roles of nuclear encoded mitochondrial proteins in development of LHON, we applied a proteomic approach to samples from affected and unaffected individuals from 3 pedigrees and from 5 unrelated controls. Two-dimensional electrophoresis followed by MS/MS analysis in the mitochondrial lysate identified 17 proteins which were differentially expressed between LHON cases and unrelated controls, and 24 proteins which were differentially expressed between unaffected relatives and unrelated controls. The proteomic data were successfully validated by western blot analysis of 3 selected proteins. All of the proteins identified in the study were mitochondrial proteins and most of them were down regulated in 11778G>A mutant fibroblasts. These proteins included: subunits of OXPHOS enzyme complexes, proteins involved in intermediary metabolic processes, nucleoid related proteins, chaperones, cristae remodelling proteins and an anti-oxidant enzyme. The protein profiles of both the affected and unaffected 11778G>A carriers shared many features which differed from those of unrelated control group, revealing similar proteomic responses to 11778G>A mutation in both affected and unaffected individuals. Differentially expressed proteins revealed two broad groups: a cluster of bioenergetic pathway proteins and a cluster involved in protein quality control system. Defects in these systems are likely to impede the function of retinal ganglion cells, and may lead to the development of LHON in synergy with the primary mtDNA mutation.

## Introduction

Leber’s hereditary optic neuropathy (LHON) [OMIM 535 000] is one of the commonest mitochondrial inherited diseases [1]. It is also one of the common causes of blindness in young men, and more than 80% of LHON patients are male [2]. As a result of degeneration of retinal ganglion cell layers, the patients usually develop bilateral acute or sub-acute, painless loss of central vision [3].

The three missense mitochondrial DNA (mtDNA) mutations 11778G>A, 14484T>C, and 3460G>A are the primary mutations responsible for 95% of LHON cases [4]. These mutations change amino acid R340H, M64V and A52T in ND4, ND6 and ND1 subunits of complex I of mitochondrial OXPHOS respectively. All of the LHON patients detected so far in Thailand carry either 11778G>A (>90% of cases) or 14484T>C [5], [6]. Though presence of a primary mutation is necessary to develop the disease, the primary mutations per se cannot explain the distinctive features of LHON [4], [7]. The incomplete penetrance of these mutations, and the disease’s greater prevalence in males, imply that carriage of a primary mutation alone is not sufficient to cause pathogenesis, and that additional genetic and environmental factors are involved [4]. Level of heteroplasmy of mutant mtDNA, mtDNA haplogroup, nuclear DNA background and various environmental factors have also been reported to influence the clinical development of LHON [4], [8]–[13]. Many previous studies have been conducted to understand these putative risk factors better, in particular to hunt for nuclear modifier genes. Nuclear genetic analyses have ranged from studies of single genes [14]–[16] to global gene expression profiling studies [17]–[19]. Given that both mitochondrial and nuclear genes encode OXPHOS subunits and that there is cross-talk between mitochondria and the nucleus, differential expression of both mitochondrial and nuclear genes is observed in various OXPHOS deficiency models [19]. The mitochondrial proteome contains approximately 1,000 proteins, 99% of which are the products of nuclear genes [20]. The coordinated expressions of imported nuclear encoded proteins with the 13 mtDNA-encoded proteins are crucial for the integrity of mitochondrial function.

In order to search for nuclear encoded mitochondrial proteins that may influence the development of LHON, this study compares the mitochondrial proteomic profiles of fibroblasts from affected and unaffected individuals in LHON families with those of unrelated controls, using 2-Dimensional Polyacrylamide Gel Electrophoresis (2-DE) and mass spectrometry.

## Materials and Methods

### Patients and Their Unaffected Relatives

The samples employed in this study were cultured dermal fibroblasts, directly obtained from 7 affected LHON patients from 3 unrelated pedigrees, 3 unaffected relatives from the 3 families (one from each), and 5 unrelated controls. All LHON cases were diagnosed by an ophthalmologist (WC) and were confirmed to be homoplasmic carriers of the 11778G>A mtDNA mutation. The 3 unaffected relatives were maternally related to the affected individuals and all of them were homoplasmic carriers of the 11778G>A mtDNA mutation (Figure S1). As a control group, five individuals with no familial history of eye diseases were recruited. They were recruited during visits to Siriraj Hospital, Bangkok, Thailand, for reasons unrelated to eye diseases or chronic metabolic diseases. The study was approved by the Ethics Committee of the Mahidol University, Faculty of Medicine, Siriraj Hospital (No. 161/2551) and the study was conducted according to the principal of the World Medical Association’s Declaration of Helsinki. Written informed consent was provided for all the samples used in the research.

### Fibroblast Cell Culture

The primary dermal fibroblasts from the affected and unaffected relatives, and the unrelated controls were cultivated and maintained at 37°C with 12% (v/v) fetal bovine serum (FBS) in Dulbecco’s modified Eagle’s medium supplemented with amphotericin B (1 µg/ml), penicillin (100 U/ml), streptomycin (100 µg/ml), 2 mM L-Glutamine, uridine 50 µg/ml [21] in humidified 5% CO_2_ atmosphere at 37°C. The medium was refreshed 3 times a week. Cells were harvested at passage 6 for mitochondrial isolation.

### Immunofluorescent Staining for Fibroblast Confirmation

For immunofluorescent staining, the cells were washed three times with PBS and fixed in 3.8% formaldehyde in PBS at room temperature for 10 minutes. After rinsing with PBS, the fixed cells were blocked with 1% BSA PBS for 30 minutes. Mouse monoclonal anti-Fibroblast surface protein (Abcam, Cambridge, USA) (1∶50) was incubated for 2 hours at room temperature. After washing with PBS for 3 times, the cells were incubated with secondary antibody (rabbit anti-mouse conjugated with FITC 1∶2,000 in 1% BSA PBS) and Hoechst-dye 33342 at a dilution of 1∶1,000 at room temperature in the dark. Then, the cells were rinsed with PBS and mounted with anti-phase solution on glass slide and visualized under fluorescent microscopy (Nikon ECLIPSE 80i, Nikon Corp.; Tokyo, Japan).

### Fibroblast Mitochondrial Isolation

Fibroblast mitochondria were isolated by differential centrifugation [22]. Before trypsinization, the cultured cells were washed with chilled PBS at least four times to make sure that there was no residual FBS. The cultured fibroblasts were then trypsinized with 0.25% trypsin-EDTA. Samples of 0.5×10^6^ cells were suspended in 1 ml of isolation buffer containing 0.25 M sucrose, 10 mM HEPES (pH 7.5) and 0.1 mM EDTA. Cell suspensions were sonicated with a probe sonicator (Bandelin Sonopuls HD 200; Bandelin electronic; Berlin, Germany) at MS 72/D (50 cycles) for 10 sec. Cell lysates were then centrifuged twice at 1,000 *g* for 10 min to remove cell debris and intact cells, if present. Supernatant was collected and centrifuged at 20,000 *g* for 30 min. Pellets were saved and washed with the buffer containing 0.25 M sucrose and 10 mM HEPES (pH 7.5) and centrifuged again at 20,000 *g* for 20 min. As a final step, the mitochondrial pellets were washed with PBS and centrifuged at 20,000 *g* for 10 min. The whole extraction procedure was performed at 4°C. The mitochondrial pellets were lyzed by Laemmli or 2-DE buffer depending on the subsequent step of the experiment, and the lyzed mitochondrial fractions were kept at −20°C until use.

### Mitochondrial Fraction Purity Evaluation

To confirm the purity of the extracted mitochondrial fractions, mitochondrial proteins and the proteins from the whole cell lysate were resolved by western blot analysis, using mitochondrial- and other organelle-specific markers. The mitochondrial pellets or the primary fibroblasts subsequently used for the Western Blot experiments were lyzed by 2x Laemmli buffer containing 4% SDS, 10% 2-mercaptoehtanol, 20% glycerol, and 0.125 M Tris HCl without bromophenol blue [23]. The protein samples were boiled for 5 min at 95°C. In each western blot analysis, 20 µg of total proteins from each sample were loaded. The proteins were resolved by 3.7% SDS-PAGE (stacking gel) and 12% SDS-PAGE (resolving gel) at 150 V for two and a half hours by vertical gel electrophoresis. The resolved proteins in the gel were then electro-transferred to nitrocellulose membrane using a semidry transfer method (Bio-Rad; Hercules, CA) for 80 min with a constant current of 75 mA. Non-specific binding to the membrane was blocked with 5% skimmed milk in PBS for 1 hr. The blocked membranes were probed with the desired specific primary antibodies in 1% skimmed milk or 1% Bovine Serum Albumin in PBS for overnight at 4°C at concentrations according to manufacturers’ instructions. Membranes were washed with PBS for 3 times (5 min per wash) and further incubated with the required secondary antibodies conjugated with horse radish peroxidase (Dako, Glostrup, Denmark), in 1% skimmed milk in PBS or 1% BSA in PBS. Incubations with the secondary antibodies were carried out at room temperature for 1 hr, with the concentrations of the secondary antibodies half of those used for the primary antibodies. Bands were visualized by Super Signal West Pico chemiluminescence substrate (Pierce Biotechnology Inc.; Rockford, IL, USA). The following primary antibodies were used: rabbit polyclonal anti-voltage dependent anion selective channel protein 1 (VDAC-1), a mitochondrial marker (Abcam, Cambridge, USA; ab28777); rabbit polyclonal anti-lysosomal associated membrane protein-2 (LAMP-2), a lysosomal marker (Abcam, Cambridge, USA; ab37024); rabbit polyclonal anti-Calnexin, an endoplasmic reticulum marker (Abcam, Cambridge, USA; ab22595); mouse monoclonal anti-c-Jun, a nuclear marker (Santa Cruz Biotechnology, Inc, sc166540) and mouse monoclonal anti-α-tubulin, a cytoplasmic marker (Santa Cruz Biotechnology, Inc, sc23948).

### Mitochondrial Proteomics Profile Analyses

#### Two-Dimensional Electrophoresis

Mitochondrial pellets derived from cultured primary fibroblasts of the 7 individuals with LHON, the 3 unaffected relatives and the five unrelated controls were lyzed with a lysis buffer containing 7 M urea, 2 M thiourea, 2% CHAPS, 120 mM DTT, 40 mM Tris, and 2% ampholyte (pH 3–10), then incubated at 4°C for 30 min. Protein concentration was determined by the Bradford method [24]. Samples of 100 µg of total mitochondrial protein from each individual were mixed with rehydration buffer (7 M urea, 2 M thiourea, 2% CHAPS, 120 mM DTT, 40 mM Tris-base, 2% ampholytes (pH 3–10) and a trace of bromophenol blue) to make the final volume of 150 µl. The samples were rehydrated onto 7 cm immobilized pH gradient DryStrips (non-linear pH gradient of 3–10; GE Healthcare, Uppsala, Sweden) at room temperature for 16 hr. IPG strips were focused in an Ettan IPGphor II IEF System (GE health care) at 20°C using a stepwise mode to reach 9083 Vh.

After isoelectric focusing, the strips were equilibrated in two different equilibration buffers at room temperature, for 15 min each time. The first equilibration buffer contained 6 M urea, 130 mM DTT, 112 mM Tris-base, 4% SDS, 30% glycerol, and 0.002% bromophenol blue, and the second equilibration buffer contained the same components except 135 mM iodoacetamide instead of DTT. The strips were further loaded on 13% polyacrylamide gel and resolved using a SE260 mini Vertical Electrophoresis Unit (GE Health Care) at 150 V for approximately 2 hr. The separated proteins were fixed with 10% methanol and 7% acetic acid for 30 min. The fixed solution was then removed and the gels were stained with 20 ml of Deep Purple fluorescence stain (GE Healthcare) overnight on a continuous gentle rocker. Gel images were taken using a Typhoon 9200 laser scanner (GE Healthcare).

#### Analysis of Protein Spots

Detection and matching of spots on gel images and analysis of protein spots was performed using ImageMaster 2D Platinum software from GE Health care. Parameters used for spot detection were: minimal area of 10 pixels, smooth factor of 2 and saliency of 2. The gel containing all of the spots and with the greatest number of spots of all the gels was classed as the reference gel. It was used to check for the presence and differential expression of proteins among gels. Background subtraction was performed, and the intensity volume of each spot was normalized with total intensity volume (summation of the intensity volumes obtained from all spots within the same 2-D gel). Intensity volumes of individual spots from each gel were subjected to statistical analysis to compare between the three groups of samples in the study. Protein spots that were differentially expressed at significant levels were subjected to in-gel tryptic digestion for identification by mass spectrometry.

The significantly differentially expressed spots of proteins were manually excised from the gels. The excised gel pieces were washed twice with 200 µL of 50% acetonitrile (ACN)/25 mM NH_4_HCO_3_ buffer (pH 8.0) at room temperature for 15 min, and then washed once with 200 µL of 100% ACN. After washing, the solvent was removed and the gel pieces were dried. The dried gel plugs were then rehydrated with 10 µL of 1% (w/v) trypsin in 25 mM NH_4_HCO_3_. After rehydration at 37°C for 30 min, the gel pieces were crushed and further incubated with 1% (w/v) trypsin at 37°C for at least 16 hr. Peptides were subsequently extracted twice with 50 µL of 50% ACN/5% trifluoroacetic acid; the extracted solutions were then combined and dried with the SpeedVac concentrator. The peptide pellets were resuspended with 10 µL of 0.1% TFA and concentrated. The peptide solution was then washed with 10 µL of 0.1% formic acid by drawing up and expelling the washing solution three times. The peptides were eluted with 5 µL of 75% ACN/0.1% formic acid.

#### Protein Identification by Mass Spectrometry (Q-TOF MS and/or MS/MS)

Digested peptides were injected into a Magic C18 column (Michrom Bioresources, Inc., CA, USA), which was connected to the MAGIC 2002 (Michrom Bioresources, Inc., CA, USA) high-performance liquid chromatography (HPLC) system. The solvent composition of the mobile phase was programmed to change in 50-min cycles with varying mixing ratios of solvent A (2% v/v CH_3_CN and 0.1% v/v HCOOH) to solvent B (90% v/v CH_3_CN and 0.1% v/v HCOOH) at flow rate of 1 µL/min using a MAGIC Variable Splitter. Thereafter, the peptides were eluted with a linear gradient from 0% to 50% solvent B. Purified peptides were introduced from HPLC to Q-star (Applied Biosystems, Foster City, CA, USA), a hybrid quadrupole time-of-flight mass spectrometer, through a FortisTip (AMR, Tokyo, Japan). The MS/MS data were extracted and proteins were identified using the MASCOT search engine (http://www.matrixscience.com), assuming that peptides were monoisotopic, and that fixed modifications were carbamidomethylations of cysteine residues, whereas variable modifications were oxidations at methionine residues. Only one missed trypsin cleavage was allowed, and peptide mass tolerances of 50 ppm were allowed for MS/MS ion searches. The searches were done against human proteins in the NCBI database (http://www.ncbi.nlm.nih.gov). Peptides with ion scores greater than 34 were considered as significant hits. Only the significant hits from the MS/MS peptide ion search were reported.

### Western Blot Analysis for confirmation of 2-D proteomic results

Western blot analysis was performed to validate the levels of protein expression changes identified in the proteomics profiling. 20 µg samples of the mitochondrial fractions from each sample were resolved employing the same protocol described above. Three primary antibodies were used: rabbit polyclonal HSP60 (Santa Cruz Biotechnology, Inc, sc13966), rabbit polyclonal anti-Catalase (Abcam, Cambridge, USA; ab16731) and rabbit polyclonal NDUFS1 (Abcam, Cambridge, USA; ab96428). Rabbit polyclonal anti-VDAC was used as a loading control. After incubating with the respective secondary antibodies (anti-rabbit), bands were visualized by enhanced chemiluminescence and exposed to film. The band intensities were measured using ImageJ software (http://rsbweb.nih.gov/ij/).

### Statistical Analysis

All data representing the intensity volume of the spots were reported as mean ±SEM. For comparison between 3 different groups, the data were analyzed using one-way ANOVA followed by a Post Hoc Tukey-Kramer Test (SPSS, version 18). *P-*values less than 0.05 were considered statistically significant. The false discovery rate (FDR) was determined using R statistical packages for calculating q-values and FDR-values [25], [26].

## Results

### LHON Patients and Their Unaffected Relatives

Samples from individuals in three unrelated LHON pedigrees with different mtDNA haplogroup backgrounds were employed in this study (Figure S1). The numbers of affected LHON patients recruited from pedigrees F1, F9 and F66 were two, two and three respectively. All affected individuals have been diagnosed by an ophthalmologist (WC) and have been confirmed as bearing homoplasmic mtDNA 11778G>A [9], [13], [27]. The characteristics of each patient are summarized in Table 1. The 3 unaffected relatives came from the 3 pedigrees and were maternally related to the affected individuals and also confirmed to be homoplasmic carriers of the mtDNA 11778G>A mutation. The 11778G>A mutation was not detected in the 5 control individuals recruited in this study, after performing both RFLP genotyping and Sanger sequencing in both the forward and reverse directions.

**Table 1:** Characteristics of LHON cases and unaffected relatives recruited in the study.

**Subjects**	**Gender**	**Age at present** **(years)**	**Age of onset** **(years)**	**Age at skin** **biopsy (years)**	**Fibroblast Passage** **used**	**G11778A**	**Haplogroup**	**mtDNA/nDNAratio** *	**References**
**Affected LHON**									
**Pedigree F1**									[13], [27]
**(A1)**	M	25	7	20	P6	homoplasmy	M7b1a1e1	0.84	
**(A2)**	M	26	14	21	P6	homoplasmy	M7b1a1e1	0.79	
**Pedigree F9**									[13], [27]
**(A3)**	M	30	9	24	P6	homoplasmy	C7a1	0.82	
**(A4)**	M	49	12	43	P6	homoplasmy	C7a1	0.85	
**Pedigree F66**									[9]
**(A5)**	M	42	36	36	P6	homoplasmy	M13c	0.85	
**(A6)**	M	37	18	33	P6	homoplasmy	M13c	0.68	
**(A7)**	M	54	20	50	P6	homoplasmy	M13c	0.85	
**Unaffected LHON**									
**Pedigree F1 (U1)**	M	30		25	P6	homoplasmy	M7b1a1e1	0.72	[13], [27]
**Pedigree F9 (U2)**	M	21		15	P6	homoplasmy	C7a1	0.47	[13], [27]
**Pedigree F66 (U3)**	F	49		45	P6	homoplasmy	M13c	0.66	[9]
**Controls**									
**C1**	M	43		37	P6	NO		0.51	
**C2**	M	26		20	P6	NO		0.51	
**C3**	F	46		40	P6	NO		0.66	
**C4**	F	52		46	P6	NO		0.74	
**C5**	M	58		52	P6	NO		0.65	

*MtDNA/nuclear DNA from fibroblast of each individual was measured according to Pejznochova M, 2008 [62] by amplification of *ND5* gene in mitochondrial genome from position 13466–13650 (GenBank sequence NC_012920 gi:251831106), and *PARL* gene from nuclear genome from 16912–17165 (GenBank sequence: NC_000003.12 gi:224589815). Real-time PCR amplification was performed on Bio-Rad CFX 96 thermo cycler and the threshold cycle (Ct) was obtained. MtDNA/nuclear DNA ratio was calculated from the Ct (mtDNA)/Ct (nDNA) ratio. The increasing ratio shows decrease in amount of mtDNA per cell [62].

### Confirmation of the purity of Fibroblasts

**Figure 1:**
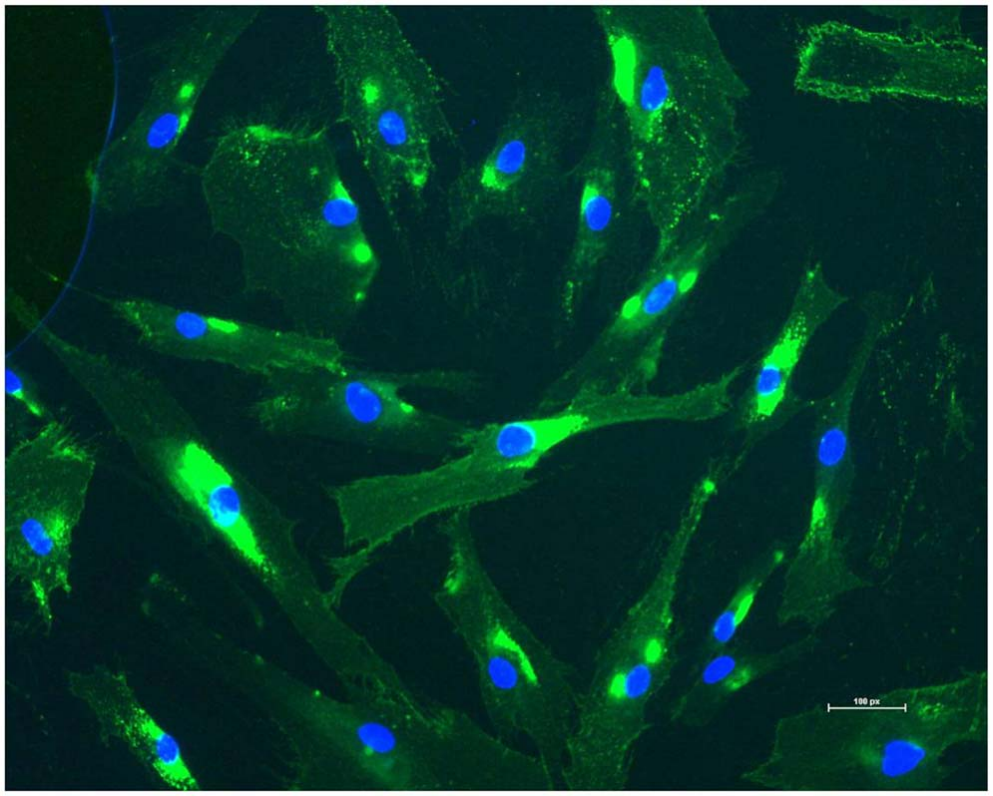
Assessment of the purity of fibroblasts from a cultured skin biopsy. Fibroblast surface protein (FSP) was used as a marker in immunofluorescence of the cultured fibroblasts obtained directly obtained from the skin biopsy. The green represents fibroblasts and the nucleus was stained with Hoechst-dye 33342 which shows blue.

One of the fibroblast cultures from each of the skin biopsies was randomly selected and tested with anti-fibroblast surface protein which is specific to fibroblasts [28]. Figure 1 shows the results of this immunofluorescent staining with DNA staining Hoechst-dye 33342, which indicated that almost every stained cell had a positive signal for fibroblast surface protein. This confirmed that the fibroblast cultures were pure and uncontaminated by other cell types.

### Mitochondrial enrichment and purity

**Figure 2:**
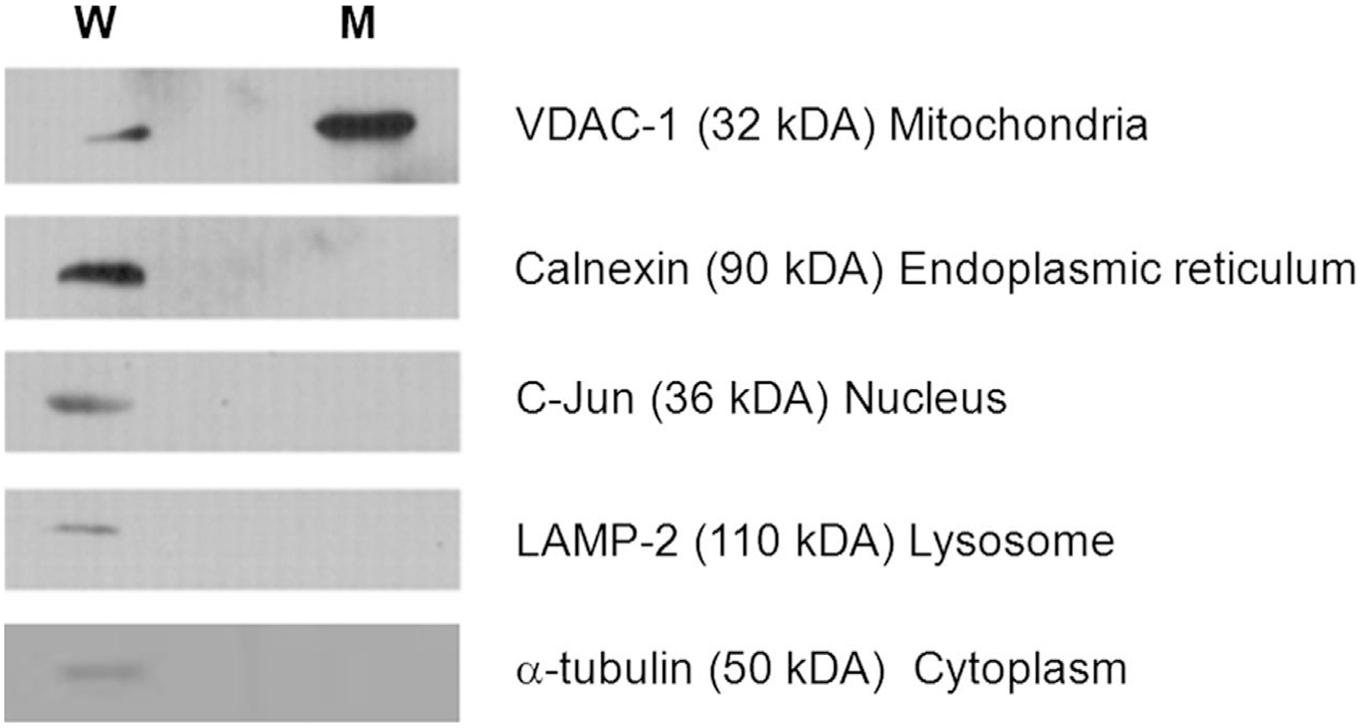
Western blot analyses for assessment of mitochondrial enrichment and purity. 20 µg of mitochondrial lysate and whole cell lysate from fibroblasts were separated by 12% SDS-PAGE gel and checked with specific antibodies against various sub-cellular organelles. (W = whole cell lysate; M = mitochondrial enriched fraction) (The same membrane for each cell type was stripped and probed with subsequent antibodies.).

Enrichment and purity of the mitochondrial fraction was assessed by immunoblotting, using markers specific for mitochondria and other organelles. Figure 2 demonstrates that the mitochondrial marker VDAC-1 was highly enriched in the mitochondrial fraction compared with the whole primary fibroblast lysates. In addition, the markers for endoplasmic reticulum, nucleus, lysosome and cytosol were absent or minimal in the mitochondrial enriched fraction. The mitochondrial fraction was highly enriched and with minimal level of non-mitochondrial contamination, and hence it was suitable for further analysis with 2-DE.

### Mitochondrial Proteomics Profile Analyses

#### 2-D PAGE comparisons of mitochondrial fraction between fibroblasts from LHON cases, unaffected relatives and unrelated controls

**Figure 3:**
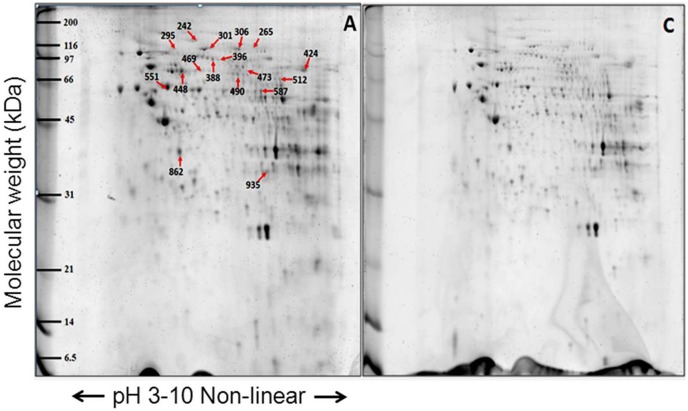
Representative proteome map of differentially expressed proteins from the mitochondrial fractions of fibroblasts from (A) LHON cases (n = 7) and (C) controls (n = 5). Equal amounts of proteins (100 µg) from each fibroblast sample were resolved by 2-DE. The numbers indicate the spot IDs of proteins whose expression levels differ significantly between the fibroblasts of LHON cases and controls.

**Figure 4:**
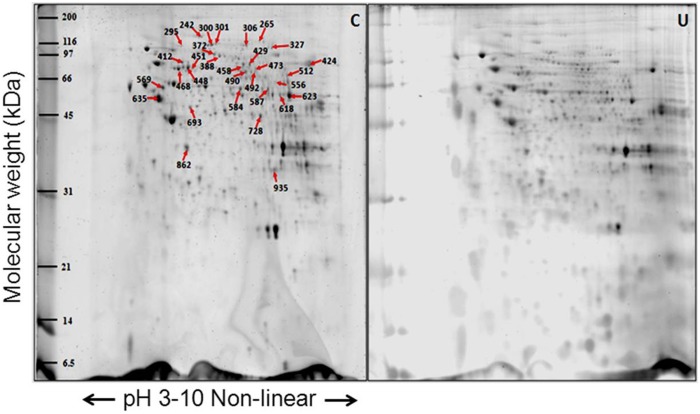
Representative proteome map of differentially expressed proteins from mitochondria fraction between fibroblasts from (U) the unaffected LHON individuals (n = 3) and (C) the controls (n = 5). Equal amounts of proteins (100 µg) from each sample were resolved by 2-DE. The numbers indicate the spot IDs of proteins whose expression levels differ significantly between the fibroblasts of unaffected LHON individuals and controls. Some spots in the same horizontal row showed the same proteins. For example, spot IDs 300 and 301 were identified as LONP1 and IDs 448, 451, 468 as catalase.

**Figure 5:**
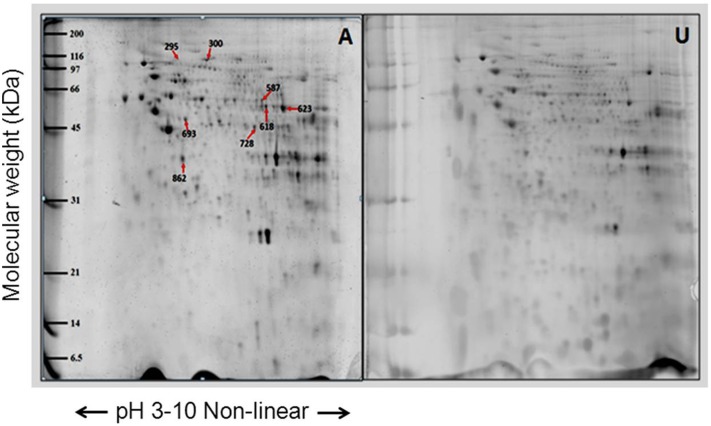
Representative proteome map of differentially expressed proteins from the mitochondrial fractions of fibroblasts from (A) LHON cases (n = 7) and (U) unaffected LHON individuals (n = 3). Equal amounts of proteins (100 µg) from each sample were resolved by 2-DE. The numbers indicate the spot IDs of proteins whose expression levels differ significantly between the LHON cases and their unaffected relatives.

The proteins from the mitochondrial enriched fraction of the primary fibroblasts were analyzed by 2-DE using pH 3–11 non-linear pH gradient strips for the first dimension and 13% SDS-PAGE for the second dimension. The individual 2-D mitochondrial proteome profiles from fibroblasts of individuals with LHON (n = 7), unaffected relatives (n = 3) and unrelated controls (n = 5) showed virtually identical spot patterns (Figure 3, 4 and 5). Approximately 800 protein spots were visualized on each gel. However, spot matching analysis revealed distinct differences in the proteomic profiles of the three groups of samples.

Interestingly, the majority of all differential-intensity spots were lower intensity in the LHON cases than in the unrelated controls. The degrees of fold-change between the cases and the unrelated controls ranged from 0.47 to 2.05. Meanwhile, the fold-changes between the unaffected relatives and the unrelated controls ranged from 0.28 to 3.90, and those between cases and unaffected relatives ranged from 1.5 to 2.2 fold. In total 61 differential intensity spots were identified in the three pairwise comparisons. These were excised from the gels, and their corresponding proteins were identified by MS or MS/MS analysis.

#### Proteins which were differentially expressed in the fibroblast of LHON cases, unaffected relatives and unrelated controls

Some sets of spots identified by MS/MS analysis in the same horizontal row were found to correspond to the same protein. Of the 61 differential-intensity spots, 33 spots representing 27 unique proteins were identified. Different isoforms of the protein mitofilin were represented by different spots in the gel. Seventeen proteins were differentially expressed between LHON cases and unrelated controls, and 24 proteins were differentially expressed between unaffected relatives of cases and unrelated controls (Table 2 and 3). Seven proteins were differently expressed between the LHON cases and their unaffected relatives (Table 4). An assessment of mitochondrial localization using the MitoMiner database [29] indicated that most of these identified proteins were mitochondrial resident proteins, and some had functional associations with mitochondria, though some were also localized in other compartments of the cell (Table 5).

**Table 2:** Summary of significantly altered proteins between the LHON cases and the control group.

Spot ID	Protein name	Gene*	NCBI ID	Identification scores(MS/MS)	%Cov(MS/MS)	number ofmatched peptides(MS/MS)	emPAI	p*I*	MW (kDa)	Intensity (Mean ± SEM)		Fold changeAffected/Control	*P* #	FDR
										Affected (n = 7)	Control (n = 5)			
242	Leucine-rich PPRmotif-containingprotein	LRPPRC	gi|31621305	1341	54	72	2.22	5.81	159.00	0.0984±0.0104	0.1746±0.0225	0.48	0.008	0.009
295	Major vaultprotein	MVP	gi|19913410	743	46	41	1.68	5.34	99.55	0.0519±0.0043	0.0856±0.0062	0.61	0.001	0.003
301	Lon proteasehomolog	LONP1	gi|21396489	1087	50	50	2.53	6.01	106.94	0.0414±0.0054	0.0861±0.0063	0.48	<0.001	<0.003
306	Vinculin isoformVCL	VCL	gi|4507877	1361	59	64	4.01	5.83	117.22	0.1089±0.0068	0.1704±0.0182	0.64	0.005	0.007
388	Transmembraneprotein – mitofilin	IMMT	gi|1160963	1174	67	57	5.97	6.15	83.89	0.1586±0.0121	0.2506±0.0168	0.63	0.001	0.003
396	Methylmalonyl-CoA mutase	MUT	gi|187452	522	34	21	1	6.48	83.54	0.0516±0.0051	0.0843±0.0069	0.61	0.012	0.010
424	Trifunctionalenzyme subunitalpha	HADHA	gi|20127408	1297	65	55	4.41	9.16	83.69	0.6631±0.0725	1.0434±0.1170	0.64	0.048	0.0232
448	MTHSP75	HSPA9	gi|292059	2174	68	84	11.1	5.97	74.02	0.4530±0.0536	0.7416±0.1002	0.61	0.030	0.017
469	Cellular myosinheavy chain	MYH9	gi|553596	604	24	25	0.6	5.70	155.29	0.0815±0.0054	0.1414±0.0260	0.58	0.041	0.021
473	Glycerol-3-phosphatedehydrogenase	GPD2	gi|1020315	1080	64	47	3.98	7.58	81.30	0.0341±0.0052	0.0660±0.0100	0.52	0.018	0.013
490	Succinate dehydrogenase[ubiquinone] flavoproteinsubunit	SDHA	gi|156416003	1019	57	39	2.96	7.06	73.67	0.1323±0.0105	0.2242±0.0338	0.59	0.018	0.013
512	Very long-chainspecific acyl-CoAdehydrogenase	ACADVL	gi|4557235	963	64	38	4.14	8.92	70.35	0.0898±0.0129	0.1423±0.0096	0.63	0.018	0.013
551	60 kDa heatshock protein	HSPD1	gi|31542947	2360	77	99	14.12	5.7	61.19	0.6804±0.5417	0.9936±0.0941	0.68	0.037	0.020
556	Catalase	CAT	gi|4557014	694	55	25	2.54	6.9	59.95	0.0648±0.0095	0.1004±0.0062	0.65	0.025	0.015
587	Dihydrolipoamide DehydrogenaseAnd DihydrolipoamideDehydrogenase-BindingProtein (Didomain) SubcomplexOf Human PyruvateDehydrogenase Complex.	DLD	gi|83753870	563	51	28	2.86	7.95	54.18	0.1873±0.0124	0.2402±0.0184	0.78	0.047	0.023
862	Pyruvate dehydrogenase E1 component subunit alpha	PDHA1	gi|33357460	572	36	17	1.87	8.35	43.30	0.6067±0.0425	0.7545±0.0185	0.80	0.039	0.020
935	Electron transfer flavoprotein subunit alpha, mitochondrial isoform a	ETFA	gi|4503607	343	63	17	3.47	8.62	33.42	0.2686±0.0210	0.3711±0.0162	0.72	0.007	0.008

*Gene name according to HUGO Gene Nomenclature Committee.

# indicates *P* value based on Post Hoc Tukey Test after one way ANOVA and the value less than 0.05 is considered significant in both tests.

**Table 3:** Summary of significantly altered proteins between the unaffected LHON individuals and the control group.

Spot ID	Protein name	Gene*	NCBI ID	Identification scores (MS/MS)	%Cov (MS/MS)	number of matched peptides (MS/MS)	emPAI	p*I*	MW (kDa)	Intensity (Mean ± SEM)		Fold Change	*P* #	FDR
										Unaffected (n = 3)	Control (n = 5)	Unaffected/control		
242	Leucine-rich PPRmotif-containingprotein	LRPPRC	gi|31621305	1341	54	72	2.22	5.81	159.00	0.0832±0.0076	0.1746±0.0225	0.48	.010	0.009
265	2–Oxoglutaratedehydrogenase	OGDH	gi|51873036	1061	48	51	1.96	6.39	115.94	0.0228±0.0041	0.0769±0.0152	0.30	.033	0.018
295	Major vaultprotein	MVP	gi|19913410	743	46	41	1.68	5.34	99.55	0.0273±0.0069	0.0856±0.0062	0.32	.000	0.001
300	Lon proteasehomolog	LONP1	gi|21396489	1004	44	45	2.17	6.01	106.94	0.1572±0.0320	0.3181±0.0222	0.49	.023	0.015
301	Lon proteasehomolog	LONP1	gi|21396489	1087	50	50	2.53	6.01	106.94	0.0292±0.0055	0.0861±0.0063	0.34	.000	0.002
306	Vinculin isoformVCL	VCL	gi|4507877	1361	59	64	4.01	5.83	117.22	0.0700±0.0014	0.1704±0.0182	0.41	.001	0.003
327	Glucosidase 2subunit betaisoform 2	PRKCSH	gi|194382324	522	32	22	1.73	4.35	61.07	0.0588±0.0127	0.1365±0.0117	0.43	.018	0.013
372	Mitochondrialinner membraneprotein isoform 1	IMMT	gi|154354964	1243	67	50	3.93	6.08	84.03	0.1115±0.0020	0.1752±0.0121	0.64	.008	0.009
388	Transmembraneprotein – mitofilin	IMMT	gi|1160963	1174	67	57	5.97	6.15	83.89	0.1312±0.0129	0.2506±0.0168	0.52	.001	0.003
412	NADH dehydrogenase(ubiquinone)Fe-S protein 1	NDUFS1	gi|21411235	1422	63	52	4.55	5.8	80.42	0.1077±0.0112	0.2194±0.0221	0.49	.023	0.015
424	Trifunctionalenzyme subunitalpha	HADHA	gi|20127408	1297	65	55	4.41	9.16	83.69	0.4438±0.1842	1.0434±0.1170	0.43	.013	0.011
429	Mitochondrialinner membraneprotein isoform 2	IMMT	gi|154354962	1079	66	57	5.69	6.15	83.90	0.1016±0.0099	0.2454±0.0421	0.41	.039	0.020
448	MTHSP75	HSPA9	gi|292059	2174	68	84	11.1	5.97	74.02	0.3171±0.0471	0.7416±0.1002	0.43	.012	0.010
451	MTHSP75	HSPA9	gi|292059	1347	60	57	6.42	5.97	74.02	0.1279±0.0204	0.2277±0.0045	0.56	.024	0.015
458	Propionyl-CoA carboxylase	PCCA	gi|296366	238	23	16	0.26	7.24	80.64	0.0326±0.0031	0.0723±0.0113	0.45	.051	0.024
468	MTHSP75	HSPA9	gi|292059	1068	58	44	4.02	5.97	74.02	0.1996±0.0133	0.4933±0.0143	0.40	.010	0.009
473	Glycerol-3-Phosphatedehydrogenase	GPD2	gi|1020315	1080	64	47	3.98	7.58	81.30	0.0251±0.0059	0.0660±0.0100	0.38	.015	0.012
490	SuccinateDehydrogenase[ubiquinone]Flavoproteinsubunit	SDHA	gi|156416003	1019	57	39	2.96	7.06	73.67	0.0628+0.0101	0.2242±0.0338	0.28	.002	0.004
492	Glutaminasekidney isoform	GLS2	gi|156104878	526	42	26	1.65	7.85	74.24	0.0210±0.0055	0.0683±0.0134	0.31	.021	0.014
512	Very long-chainspecific acyl-CoA dehydrogenase	ACADVL	gi|4557235	963	64	38	4.14	8.92	70.35	0.0609±0.0078	0.1423±0.0096	0.43	.004	0.006
556	Catalase	CAT	gi|4557014	694	55	25	2.54	6.9	59.95	0.0456±0.0057	0.1004±0.0062	0.45	.007	0.008
569	Vimentin	VIM	gi|62414289	1142	67	49	9.47	5.05	53.68	0.3070±0.0379	0.6188±0.0544	0.50	.015	0.012
584	Glutaminasekidney isoform	GLS2	gi|114582297	1015	55	40	3.86	8.09	66.22	0.155±0.0134	0.3116±0.0314	0.50	.004	0.006
587	DihydrolipoamideDehydrogenase AndDihydrolipoamideDehydrogenase-Binding Protein(Didomain)Subcomplex OfHuman PyruvateDehydrogenaseComplex.	DLD	gi|83753870	563	51	28	2.86	7.95	54.18	0.1219±0.0050	0.2402±0.0184	0.51	.001	0.003
618	GlutamateDehydrogenase-Apo Form	GLUD1	gi|20151189	743	65	40	5.02	7.66	56.32	0.1841±0.0300	0.3760±0.0191	0.49	.010	0.009
623	ATP synthasesubunit alpha	ATP5A1	gi|4757810	1379	65	79	7.26	9.07	59.67	0.5828±0.1452	1.1951±0.0176	0.49	.003	0.004
635	Oxidized Beta-Actin	ACTB	gi|146386601	1118	76	82	14.45	5.29	42.00	0.9409±0.0640	1.8908±0.0496	0.50	.030	0.017
693	Ubiquinol-cytochrome creductase core Iprotein	UQCRC1	gi|515634	382	52	22	2.38	5.94	52.59	0.1595±0.0194	0.2847±0.0288	0.56	.009	0.009
728	Ubiquinol-cytochrome creductase core Iprotein	UQCRC1	gi|515634	824	48	22	1.76	5.94	53.27	0.1948±0.0222	0.4530±0.0278	0.43	<.001	0.002
862	PyruvateDehydrogenaseE1 componentsubunit alpha	PDHA1	gi|33357460	572	36	17	1.87	8.35	43.30	0.3537±0.0487	0.7545±0.0185	0.47	<.001	0.001
935	Electron transferFlavoproteinsubunit alpha	ETFA	gi|4503607	343	63	17	3.47	8.62	33.42	0.2122±0.0209	0.3711±0.0162	0.57	.002	0.004

*Gene name according to HUGO Gene Nomenclature Committee.

# indicates *P* value based on Post Hoc Tukey Test after one way ANOVA and the value less than 0.05 is considered significant in both tests.

**Table 4:** Summary of significantly altered proteins between the LHON cases and the unaffected relatives.

Spot ID	Protein name	Gene*	NCBI ID	Identification scores (MS/MS)	%Cov (MS/MS)	number of matched peptides (MS/MS)	emPAI	p*I*	MW (kDa)	Intensity (Mean ± SEM)		Fold change Affected/unaffected	*P* #	FDR
										Affected (n = 7)	Unaffected (n = 3)			
295	major vaultprotein	MVP	gi|19913410	743	46	41	1.68	5.34	99.55	0.0519±0.0043	0.0273±0.0069	1.90	.033	0.018
300	lon proteasehomolog	LONP1	gi|21396489	1004	44	45	2.17	6.01	106.94	0.3006±0.0325	0.1572±0.0320	1.91	.031	0.018
587	DihydrolipoamideDehydrogenaseAnd DihydrolipoamideDehydrogenase-Binding Protein(Didomain)Subcomplex OfHuman PyruvateDehydrogenaseComplex.	DLD	gi|83753870	563	51	28	2.86	7.95	54.18	0.1873±0.0124	0.1219±0.0050	1.54	.037	0.020
618	GlutamateDehydrogenase-Apo Form	GLUD1	gi|20151189	743	65	40	5.02	7.66	56.32	0.3220±0.0353	0.1841±0.0300	1.75	.046	0.023
623	ATP synthasesubunit alpha	ATP5A1	gi|4757810	1379	65	79	7.26	9.07	59.67	0.9932+0.0873	0.5828±0.1452	1.70	.025	0.015
693	ubiquinol-cytochrome creductase core Iprotein	UQCRC1	gi|515634	382	52	22	2.38	5.94	52.59	0.2762±0.0135	0.1595±0.0194	1.73	.010	0.009
728	ubiquinol-cytochrome creductase core Iprotein	UQCRC1	gi|515634	824	48	22	1.76	5.94	53.27	0.4247±0.0285	0.1948±0.0222	2.18	.001	0.003
862	PyruvatedehydrogenaseE1 componentsubunit alpha	PDHA1	gi|33357460	572	36	17	1.87	8.35	43.30	0.6067+0.0425	0.3537±0.0487	1.72	.004	0.006

*Gene name according to HUGO Gene Nomenclature Committee.

# indicates *P* value based on Post Hoc Tukey Test after one way ANOVA and the value less than 0.05 is considered significant in both tests.

**Table 5:** Functional categories and sub-cellular localizations of proteins identified in the study based on the MitoMiner Database and Nextprot.

Proteins	Localization
**Intermediary metabolism: TCA cycle and Carbohydrate Metabolism**	
2-oxoglutarate dehydrogenase, mitochondrial isoform 1 precursor	M
Glycerol-3-phosphate dehydrogenase	M
Dihydrolipoyl dehydrogenase	M
Pyruvate dehydrogenase E1 component subunit alpha	M
**Intermediary metabolism: Fatty acid Catabolism**	
Methylmalonyl-CoA mutase	M
Trifunctional enzyme subunit alpha	M
Very long-chain specific acyl-CoA dehydrogenase	M
Propionyl-CoA carboxylase	M
**Intermediary metabolism: Amino acid Metabolism**	
Glutaminase	M
Glutamate dehydrogenase 1	M
**Subunits of oxidative phosphorylation and electron transport function**	
Succinate dehydrogenase [ubiquinone] flavoprotein subunit	M
Cytochrome b-c1 complex subunit 1	M
Electron transfer flavoprotein subunit alpha	M
ATP synthase subunit alpha	M
NADH dehydrogenase (ubiquinone) Fe-S protein 1, 75 kDa (NADH-coenzyme Q reductase)	M
**Cristae remodeling**	
Mitochondrial inner membrane protein	M
**Mitochondrial Gene expression**	
Leucine-rich PPR motif-containing protein	M
**signal transduction**	
Major vault protein	N, M
Glucosidase 2 subunit beta	ER
**Protein stability and degradation of protein**	
Lon protease homolog	M
Stress-70 protein, mitochondrial/Heat shock 70 kDa protein 9/MTHSP75	M, C
60 kDa heat shock protein, mitochondrial	M, C
**Anti-oxidant enzymes**	
Catalase	P, MI
**cytoskeletal protein**	
Vinculin	CSK
β-actin	CSK
Vimentin	CSK
**Others**	
Myosin-9/Cellular myosin heafvy chain-type A	C, M

M = Mitochondria, MI = Mitochondrial intermembrane space, ER = Endoplasmic Reticulum, C = Cytoplasm, N = Nucleus, CSK = cytoskeleton.

Functions of the proteins were assessed using Nextprot [30]. The differentially expressed proteins could be classified in several groups: (1) those involved in intermediary metabolism (of carbohydrates, lipids and amino acids); (2) subunits of OXPHOS; (3) a cristae remodeling protein; (4) a protein involved in mitochondrial gene expression; (5) signal transduction; (6) chaperonins and proteases of the protein quality control system; (7) an antioxidant enzyme and (8) cytoskeletal structure (Table 5). All of these proteins were down-regulated in the mitochondrial fibroblast proteomes of LHON patients and their unaffected relatives, compared with the unrelated controls.

However, expression levels of heat-shock protein 60, methylmalonyl-CoA mutase and myosin heavy chain were significantly different between the cases and the unrelated controls, but not between the unaffected relatives and the unrelated controls. Conversely, expression levels of 2-oxoglutarate dehydrogenase, NADH dehydrogenase (ubiquinone) Fe-S protein 1 (NDUFS1), ATP synthase subunit alpha, glutaminase, glutamate dehydrogenase, propionyl CoA carboxylase, beta-actin, glucosidase 2 subunit beta (PRKCSH) and vimentin were significantly different only between the unaffected relatives and the unrelated controls.

Expression of seven proteins differed significantly between the cases and their unaffected relatives: these were subunits of the respiratory chain such as ubiquinol cytochrome *c* reductase core I protein and ATP synthase subunits alpha; an enzyme of the TCA cycle, dihydrolipoamide dehydrogenase; one of the subunits of the pyruvate dehydrogenase complex; lon protease and major vault protein (Table 4).

### Validation of Proteomic data by Western Blot

**Figure 6:**
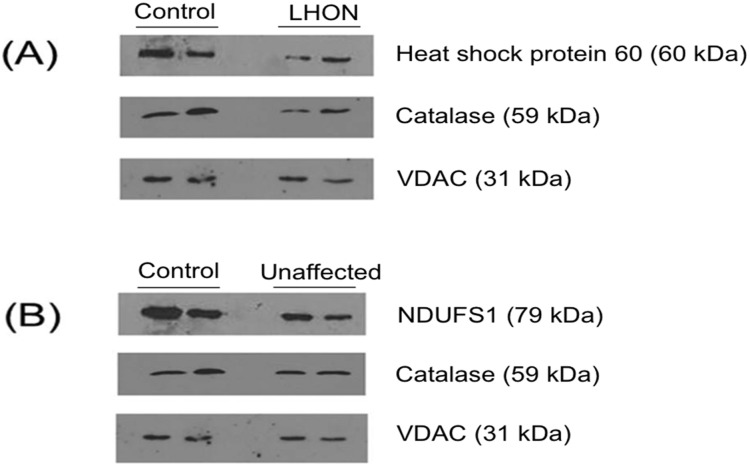
Validation of proteomic data by Western Blot analysis. These figures show: (A) The differentially expression of heat-shock protein 60 and catalase between LHON cases and controls; and (B) the differential expression of NDUFS1 and catalase between unaffected mutation carriers and controls. VDAC was used as a loading control. The two lanes represent samples from two different individuals for each group of samples.

To validate the differentially expressed proteins observed in the 2-DE proteomic analysis, three of the differentially expressed proteins - heat shock protein 60, catalase and NDUFS1 - were selected for western blot analysis. Compared with the unrelated controls, heat shock protein 60 and catalase were down-regulated in the mitochondrial fraction of the LHON cases, while catalase and NDUFS1 were down-regulated in the unaffected relatives. These differences all ran in the same direction as in the 2-DE proteomic analysis (Figure 6).

## Discussion

It is impossible to sample retinal ganglion cells, the affected target cells of this mitochondrial disease. Hence in this study we used skin fibroblasts derived from the patients, which may mirror some of the features of other post-mitotic tissues such as retinal ganglial cells. The fibroblasts were cultured directly from the skin biopsies, from LHON patients and their unaffected relatives homoplasmic for the primary mtDNA mutation 11778G>A. The purity of the skin fibroblast cultures, which may contain epithelium, reticulocytes, and adipocytes, was confirmed using anti-Fibroblast surface protein. The skin fibroblasts were then used to explore the mitochondrial proteomes of these 11778G>A carriers, comparing them with the proteomes of control individuals using 2-DE with a non-linear IPG strip of pH 3–11.

The purity and the enrichment of the mitochondrial fraction were confirmed by western blotting with organelle-specific markers. The proteomics data also confirmed this, as most of the identified proteins were mitochondrial resident proteins. No mtDNA-encoded proteins, including PARL (hydrophobic and membrane bound protein) which was previously reported [27] were identified in this study, since these proteins are highly hydrophobic [31]–[33] and 2-DE does not provide good separation of hydrophobic and membrane bound proteins [34]–[36].

**Figure 7:**
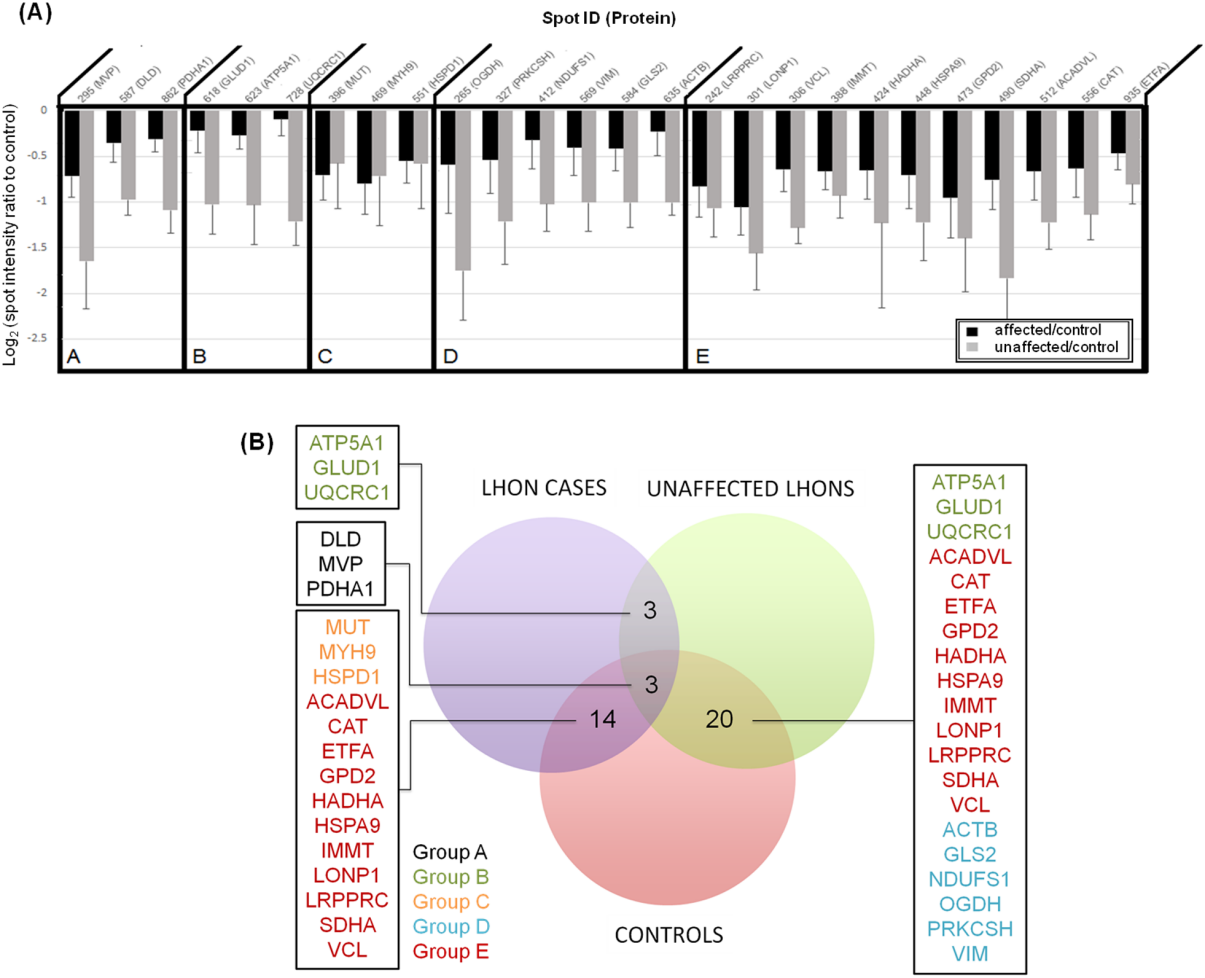
(A) A plot of log-ratio spot intensities, comparing affected individuals (black bars) and unaffected relatives (grey bars) versus controls. Log-ratios of protein spot intensities were plotted according to the method of [63]. In the case that one protein was identified in several spots, the only spot intensity that gave the highest significant value was selected for this plot. Negative changes were observed in all discoveries. Group A includes protein spots which were significantly differentially expressed in comparisons of affected and unaffected carriers, affected individuals and controls, and unaffected individuals and controls (Post Hoc Tukey Test; *P*-value<0.05). Group B includes protein spots which were significantly differentially expressed in comparisons of affected and unaffected carriers, and between unaffected carriers and controls. Changes between affected individuals and controls were not significant. Group C includes protein spots which were significantly differentially expressed only in the comparison between affected individuals and control. Group D includes protein spots which were significantly differentially expressed only in the comparison between unaffected carriers and controls. Group E includes protein spots which were significantly differentially expressed the comparisons between affected individuals and controls, and between unaffected carriers and controls. Here, the differences in expression between affected and unaffected carriers are not significantly different. **(B) Venn diagram representation of proteins identified in three comparisons derived from** **figure 7A** **.**

All of the proteins that were differentially expressed between 11778G>A carriers and unrelated controls were down-regulated in fibroblasts of 11778G>A carriers: either the LHON cases or their unaffected relatives (Figure 7). Some of the subunits of the OXPHOS complex were altered: NDUFS1 in complex I, succinate dehydrogenase in complex II, UQCRC1 in complex III and ATP5A1 in complex V were all down-regulated in fibroblasts of either the affected or the unaffected 11778G>A carriers. This was an unexpected finding since complex I defective LHON mutant cells may compensate for a lack of ATP synthesis through up-regulation of succinate/glycerol-3-phosphate dehydrogenase [37]–[39]. However, our results were in agreement with the findings of Qiang et al, where succinate/glycerol-3-phosphate driven respiration was reduced in mutant cells derived from some Chinese LHON families [40].

The proteins found to be significantly differently expressed between the cases, unaffected 11778G>A carriers and unrelated controls can be categorized functionally into 2 functional groups: bioenergetic pathways and mitochondrial protein quality control. A number of enzymes for intermediary metabolism were differentially expressed in 11778G>A mutant fibroblasts compared with unrelated controls (Table 2 and 3). Interestingly, differential expression was observed particularly in FAD-linked and NAD-linked dehydrogenases which produce reducing equivalents to feed the respiratory chain. For instance, altered expression was found for two enzymes of β-oxidation: FAD-dependent very long chain specific acyl-CoA dehydrogenase, and NAD-dependent trifunctional enzyme subunit alpha (also known as hydroxyacyl CoA dehydrogenase). And among the TCA cycle enzymes, FAD-dependent succinate dehydrogenase, NAD-dependent 2-oxoglutarate dehydrogenase, pyruvate dehydrogenase E1 component alpha subunit and dihydrolipoyl dehydrogenase were down-regulated in 11778G>A fibroblasts. Also down-regulated was glycerol-3-phosphate dehydrogenase, which is involved in transporting cytosolic reducing equivalents to the mitochondrial respiratory chain.

Given the decreased levels of metabolic and OXPHOS related proteins in mutation carriers in the present study, it is likely that aerobic respiration is particularly affected in LHON mutant fibroblasts. To further support this notion, ETFA electron transfer flavoprotein subunit alpha was down regulated in the fibroblasts of both affected and unaffected mutation carriers. This protein is important in transferring electrons from many of the mitochondrial flavoprotein dehydrogenases to the respiratory chain [41]. This bioenergetic dysfunction is consistent with previous reports of defective energy metabolism in LHON mutant fibroblasts, including reduction of complex I activity, poor respiratory capacity and reduced ATP content in mutants compared to controls [42], [43].

Several stability and transport proteins were also differentially expressed: heat shock protein 60 (HSPD1), Stress-70 protein (MTHSP75/HSPA9) and lon protease 1 (LONP1) (Table 2 and 3). HSPD1 is one of the most important chaperonins inside the mitochondrial matrix. It facilitates correct folding and prevents mis-folding of unfolded proteins formed under mitochondrial stress [44]. Mutations of HSPD1 in hereditary spastic paraplegia and MitChap60 disease highlight possible implications for neurodegenerative disease [45]–[47]. In the present study, expression of HSPD1 was reduced 1.5 fold in LHON case fibroblasts compared to unrelated controls. This reduction may have deleterious consequences if it happens in neuronal retinal ganglion cells, since they are post-mitotic and are highly susceptible to the accumulation of unfolded proteins [48]. Another chaperone Stress-70 protein (MTHSP75/HSPA9) was down-regulated in fibroblasts of both affected and unaffected mutation carriers compared with controls. In addition, HSPA1, HSPA9 and LONP1 co-occurred as differentially expressed proteins in other cellular models [49]. They are the components of the mitochondrial protein quality control system which protects the formation of the unfolded protein with chaperones and clears protein aggregates with proteases [50]. There can be detrimental consequences when the system is under-expressed, as found in the 11778G>A fibroblasts of the present study.

In addition to the proteomic changes in metabolic enzymes and the protein quality control system, some proteins controlling mitochondrial gene expression were also down-regulated in the mutation carriers: Leucine-rich pentatricopeptide repeat motif-containing protein (LRPPRC) and LONP1. LRPPRC was down-regulated in the fibroblasts of both affected and unaffected carriers of 11778G>A. It is a disease modifier in Leigh syndrome French-Canadian type [51]. Silencing of *LRPPRC* was associated with reduction of mitochondrial proteins including mitochondrial and nuclear encoded subunits of OXPHOS [52]. LONP1, a nucleoid protein, was also under-expressed in mutant cells. LONP1 binds to mtDNA, and its level influences a cell’s sensitivity to mtDNA damage [53], with potential implications for LHON pathogenesis.

Apart from proteins involved in bioenergetic pathways and mitochondrial protein quality control, catalase and mitofilin were also differentially expressed. Compared to controls, catalase was down-regulated in both affected and unaffected fibroblasts carrying 11778G>A. Though it is mainly located in the peroxisome [54], it is also associated with mitochondria in some cell types [55], [56]. Reduction or absence of catalase under oxidative stress in mitochondria can lead to the inefficient degradation of H_2_O_2_, leading to potential mitochondrial and cellular damage [57].

Mitofilin, a protein which plays a role in/influences cristae morphology and nucleoid structure, was down-regulated. Previously, down-regulation of mitofilin was observed in dopamine induced oxidative stress [58] and MPTP induced complex I inhibition [59]. Since oxidative stress and complex I inhibition are associated with LHON mutations, the down-regulation of mitofilin in 11778G>A mutant cells may have a role in LHON pathogenesis.

It is interesting to observe the similarities in fibroblast mitochondrial protein expression profiles between affected and unaffected 11778G>A carriers, in comparison with the unrelated controls. The levels of 14 (out of total 27) identified proteins were reduced both in the affected and the unaffected mutation carriers compared to the controls (Table 6). The proteins down-regulated in both groups of 11778 mutant fibroblasts were electron transfer flavoprotein, succinate dehydrogenase, dihydrolipoyl dehydrogenase, subunit of pyruvate dehydrogenase, glycerol-3-phosphate dehydrogenase concerned with electron transfer and aerobic respiration, very long chain specific acyl-CoA dehydrogenase, trifunctional plasma enzyme of β-oxidation, chaperonins such as lon protease 1, heat-shock protein-70, leucine-rich PPR motif-containing protein, mitofilin, catalase and some other proteins (Table 6). This similarity might reflect common compensatory responses cells responding similarly to the same adverse conditions, regardless of whether the cells come from affected or unaffected individuals. Since 11778G>A mutant fibroblasts have been shown to have reduced complex I activity and larger bioenergetic defects [43], these reduced levels of protein expression might be due to degradation of proteins with poor performance or to alteration of nuclear gene responses in the presence of OXPHOS deficiency. These mechanisms would be consistent with the observed results: that the cells in primary fibroblasts cultures encountering the 11778G>A mutation respond similarly to the adverse consequences of the mutation, regardless of the phenotypic status of the person who provided the biopsy. This mutation might reprogram gene expression profiles, with similar consequent physiological states of the cells driving similar proteome changes between the carriers. Similar transcriptomic profiles have been observed in previous studies, where clinically affected and unaffected tissues from the same individual have similar profiles [60].

**Table 6:** Differentially expressed proteins which show similar trends of down-regulation in the mitochondrial proteomes of both LHON cases and unaffected relatives when compared with controls.

Name of Protein	Cellular pathway or function involved
Glycerol-3-phosphatedehydrogenase	Intermediary metabolism: transferring reducing equivalents to mitochondrial repiratory chain
Dihydrolipoyl dehydrogenase	Intermediary metabolism: TCA cycle; generation of reducing equivalents for OXPHOS
Pyruvate dehydrogenaseE1 component subunit alpha	Intermediary metabolism: carbohydrate; generation of reducing equivalents for OXPHOS
Trifunctional enzymesubunit alpha	Intermediary metabolism: lipid; β-oxidation; generation of reducing equivalents for OXPHOS
Very long-chain specificacyl-CoA dehydrogenase	Intermediary metabolism: lipid; β-oxidation; generation of reducing equivalents for OXPHOS
Succinate dehydrogenase[ubiquinone] flavoprotein subunit	Intermediary metabolism: TCA cycle; generation of reducing equivalents for OXPHOS
Electron transfer flavoproteinsubunit alpha	Transfer electron from flavoprotein to OXPHOS
Mitofilin	Cristae remodeling
Lon protease 1	Protein stability and degradation
Stress-70 protein/Heat shock70 kDaprotein 9/MTHSP75	Protein stability and degradation
Catalase	Anti-oxidant enzyme
Leucine-rich PPR motif-containing protein	Mitochondrial gene expression
Vinculin	Cytoskelon
Major vault protein	Signal transduction

There are some previous studies on transcriptomal changes in LHON mutant cells using various cell types. However, they reported differing patterns of transcriptomal changes, possibly reflecting differences in susceptibility to bioenergetic derangements between cell types. For instance, transcriptomic changes from osteosarcoma-derived LHON cybrids and LHON-mutant lymphoblastoid cell lines generally showed different profiles, with the exception of nine common genes [17], [19]. Differentially expressed gene products involved in transcription and transport processes were reported in the lymphocytes of four Saudi Arab LHON patients [18]. None of these gene products was differentially expressed in our data. This could be due the employment of different cell types (fibroblasts versus osteosarcoma derived cybrids or lymphoblastoid cell lines), different sub-cellular locations that we are observing (enrichment of mitochondrial proteins in our study versus global expression profiling), or different patterns of expression at the RNA level and the protein level, because of post-translational modifications, degradation and dependence on organellar transport. Consequently, we did not find any evidence of ER stress or protein unfolding responses, which are observed in various mitochondrial diseases [19].

This study which, to the best of our knowledge, is the first study to profile mitochondrial proteomes in LHON, has some limitations. One potential limitation is the challenge of selecting well age-and sex-matched controls for cases and their related unaffected individuals. We selected pedigrees in which the unaffected individuals were well above the age of onset of their affected relatives for each particular family (Table 1). All of the three unaffected relatives, U1 from pedigree F1 (30 years old at present), U2 from pedigree F9 (21 years old at present) and U3 (49 years old at present) from pedigree F66 were above both the mean age of onset for Thai individuals (20.7±10.0 years for males and 28.6±14.6 years for females) [61], and the mean age of onset within their families, 13 years, 17 years and 32 years respectively. None of them have developed the disease, and their latest eye examinations in early 2014 were within normal limits. Another potential limitation was that not all of the unaffected individuals were of the same sex: two were male and one was female. Sex differences could potentially confound the results, especially in the comparisons between unaffected mutation carriers and controls. Nevertheless, in spite of these limitations, the results from the present study could contribute to extant understanding of proteome changes in LHON mutant cells.

In summary, our proteomic data highlight proteins that were differentially expressed between the fibroblast of LHON cases, unaffected carriers of the LHON 11778G>A primary mtDNA mutation, and normal individuals. Functional analyses of these proteins imply that bioenergetic derangements and poor protein quality control systems, both incompatible with regular functioning of retinal ganglion cells, may be involved in LHON pathogenesis. Failure to conduct proper protein folding, to assemble protein complexes properly and to prevent unfolded proteins having damaging effects on cells may lead to the onset of LHON.

## Supporting Information

Figure S1 **The three pedigrees used in the present study.** (A = LHON cases and U = unaffected relatives whose fibroblasts were used in the present study. The arrow indicates the proband of each pedigree).
(DOCX)
