# Supplementary material for: Profiling the Mitochondrial Proteome of Leber’s Hereditary Optic Neuropathy (LHON) in Thailand: Down-Regulation of Bioenergetics and Mitochondrial Protein Quality Control Pathways in Fibroblasts with the 11778G>A Mutation
Source: PLoS One. 2014 Sep 12;9(9):e106779. doi: 10.1371/journal.pone.0106779 (PMC4162555; doi:10.1371/journal.pone.0106779)
Supplement: Figure S1 — The three pedigrees used in the present study. (A = LHON cases and U = unaffected relatives whose fibroblasts were used in the present study. The arrow indicates the proband of each pedigree). (DOCX) [file pone.0106779.s001.docx]

**Figure S1. Three pedigrees used in the present study.** (A = LHON cases and U = unaffected relatives whose fibroblasts were used in the present study. The arrow indicates the proband of each pedigree.)

**Pedigree F1**

**
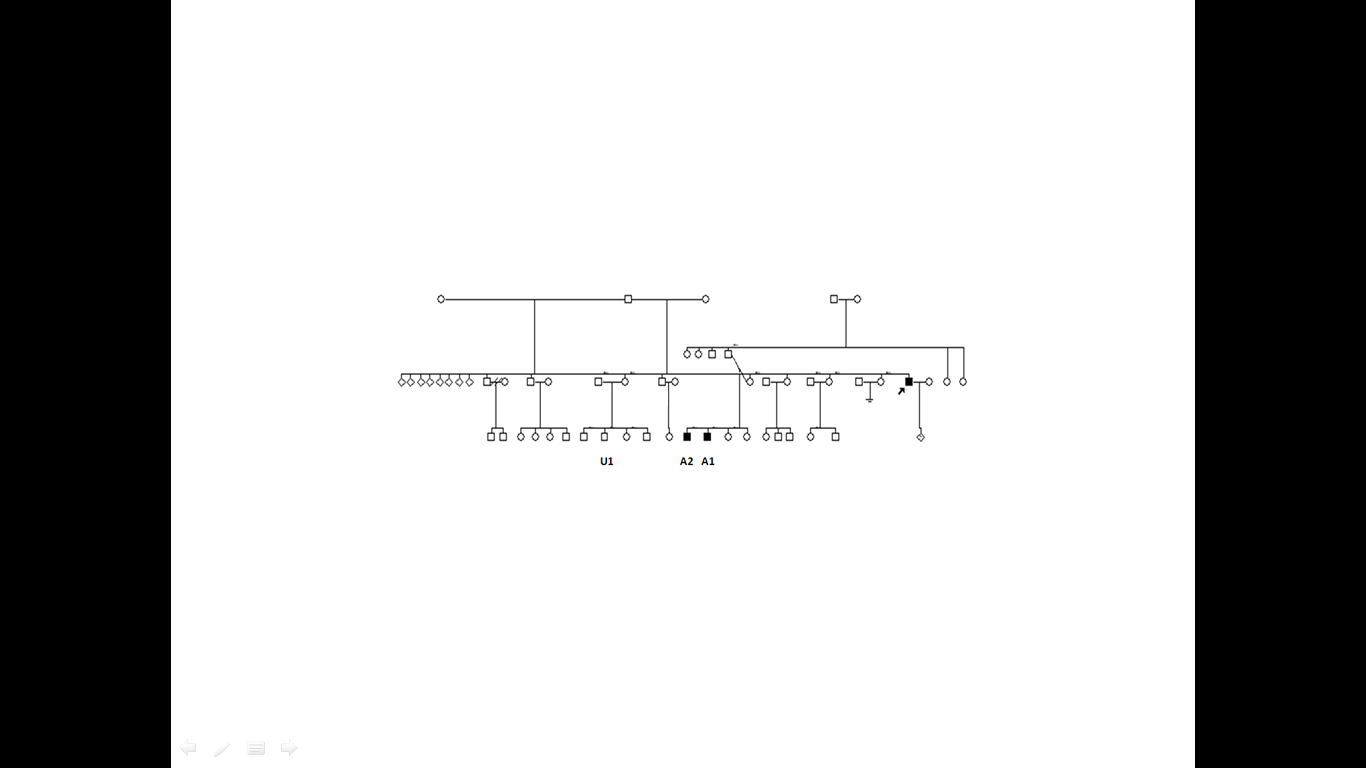
**

**Pedigree F9**

**Pedigree F66**

**
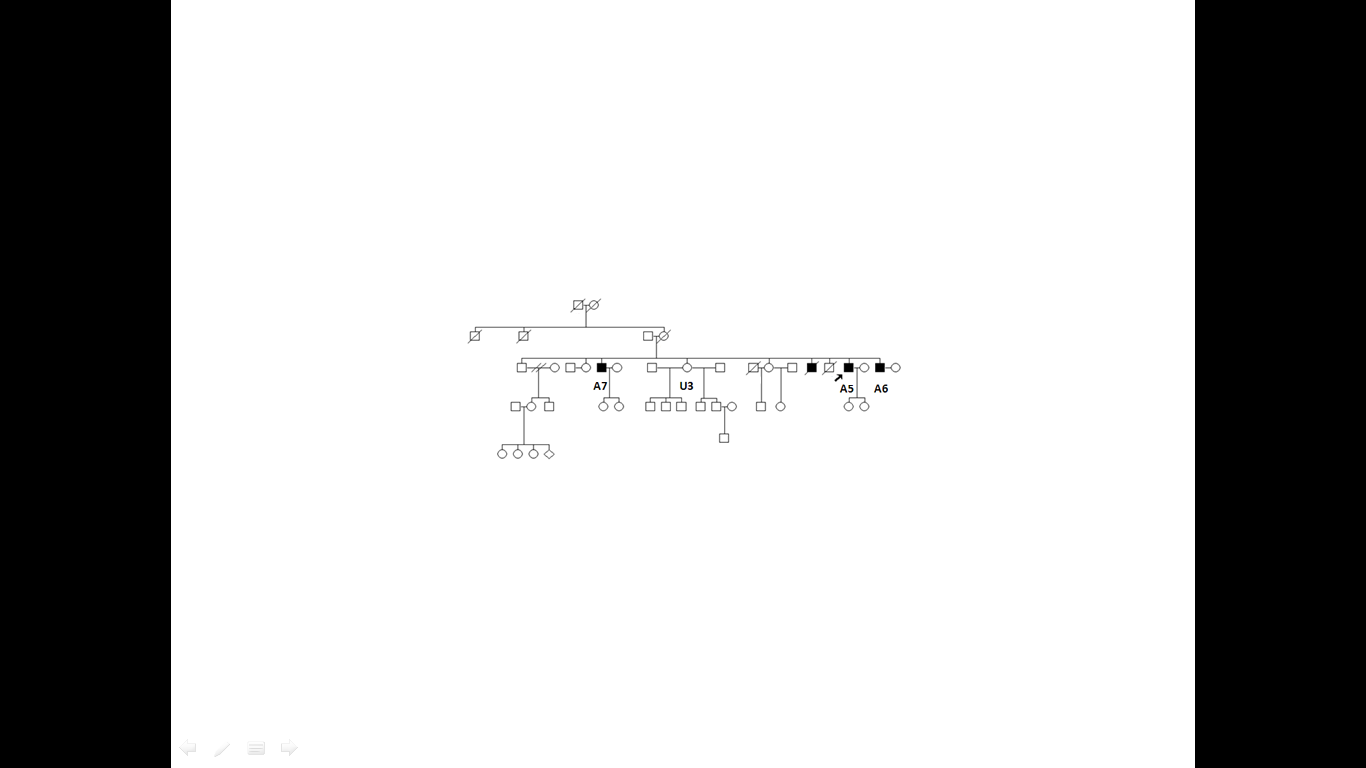
**
